# Tying Down Loose Ends in the *Chlamydomonas* Genome: Functional Significance of Abundant Upstream Open Reading Frames

**DOI:** 10.1534/g3.115.023119

**Published:** 2015-12-23

**Authors:** Frederick R. Cross

**Affiliations:** The Rockefeller University, New York, New York 10065

**Keywords:** *Chlamydomonas* genome, annotation, upstream ORFs

## Abstract

The *Chlamydomonas* genome has been sequenced, assembled, and annotated to produce a rich resource for genetics and molecular biology in this well-studied model organism. The annotated genome is very rich in open reading frames upstream of the annotated coding sequence (‘uORFs’): almost three quarters of the assigned transcripts have at least one uORF, and frequently more than one. This is problematic with respect to the standard ‘scanning’ model for eukaryotic translation initiation. These uORFs can be grouped into three classes: class 1, initiating in-frame with the coding sequence (CDS) (thus providing a potential in-frame N-terminal extension); class 2, initiating in the 5′ untranslated sequences (5UT) and terminating out-of-frame in the CDS; and class 3, initiating and terminating within the 5UT. Multiple bioinformatics criteria (including analysis of Kozak consensus sequence agreement and BLASTP comparisons to the closely related *Volvox* genome, and statistical comparison to cds and to random sequence controls) indicate that of ∼4000 class 1 uORFs, approximately half are likely *in vivo* translation initiation sites. The proposed resulting N-terminal extensions in many cases will sharply alter the predicted biochemical properties of the encoded proteins. These results suggest significant modifications in ∼2000 of the ∼20,000 transcript models with respect to translation initiation and encoded peptides. In contrast, class 2 uORFs may be subject to purifying selection, and the existent ones (surviving selection) are likely inefficiently translated. Class 3 uORFs are found in more than half of transcripts, frequently multiple times per transcript; however, they are remarkably similar to random sequence expectations with respect to size, number, and composition, and therefore may in most cases be selectively neutral.

The assembled *Chlamydomonas* reference genome is 120 Mb long, 65% GC, and very repeat-rich (Merchant *et al.* 2007; Blaby *et al.* 2014). The assembly contains 17 chromosomes (∼1–10 Mb) and a further 37 repeat-rich ‘scaffolds’ (0.1–0.8 Mb). The genome has been annotated with 19,526 transcript models including transcription starts and stops, intron/exon boundaries, and coding sequence (CDS) (Blaby *et al.* 2014), and the resulting annotated assembly is available on a public-access website (http://phytozome.jgi.doe.gov/pz/portal.html) maintained by JGI (hence ‘Phytozome’). For a subset of genes, many of these features have been verified by comparison to EST databases, as indicated on the Phytozome website. There still appears to be a need for bioinformatic methods to ‘proofread’ proposed selection of translation initiation codons in the transcript models because, as will be detailed below, potential initiation codons upstream of the reference initiator are very numerous in the annotation.

## Materials and Methods

*Chlamydomonas* sequence files were downloaded from the Phytozome website, as was the .gff3 annotation file that specifies location and strand of transcript exons and CDS. *Volvox* predicted proteome sequences were also from the Phytozome website. BLASTP (Altschul *et al.* 1990) was by a local installation of the NCBI BLAST suite. Other calculations were coded in MATLAB. Scripts and functions that will operate on fasta and gff files downloaded from Phytozome are provided in Supporting Information, File S2. The codes produce most calculations and figures, as well as the file Creinhardtii_281_v5.5_transcript_summary.mat that contains preassembled data and calculations of various kinds about all annotated *Chlamydomonas* transcripts. Code is also provided to update this .mat file in the event of a future new gff or fasta release.

## Results

### Translation start sites and 5′ untranslated open reading frames

Annotating gene content from assembled genomic sequences poses many challenges (Blaby *et al.* 2014). Consensus sequences for transcriptional initiation are uncertain, so beginnings of transcripts are uncertain. Polyadenylation/termination and RNA splicing have moderately high information-content consensus sequences, but these sequences clearly do not account for all RNA processing ‘choices’, and splicing intrinsically adds a huge number of degrees of freedom for computationally assembling translational open reading frames and associated 5′ and 3′ untranslated regions of transcripts. This process was aptly called ‘Gene modeling, or finding needles in a haystack’ (Blaby *et al.* 2014).

The genome sequence and the borders of annotated 5UT, cds, intron, and 3′ untranslated regions are available on Phytozome, allowing reassembly of the complete set of 19,526 transcript models on 17 chromosomes (and an additional 37 unassembled scaffolds) to provide sequences of all 5UT regions and associated cds. (Note: the 19,526 transcripts are derived from ∼17,000 ‘gene’ models; the extra transcripts are due to proposed alternative initiation, splicing, and or termination events. I elected to treat the transcript models as independent, since it is possible that different transcripts from some gene model might differ with respect to 5UT or other relevant features. This provides the possibility of a minor level of duplication of results for some findings; an informal evaluation suggests that this duplication is approximately randomly dispersed among functional categories.)

There are no obvious bioinformatic methods to reliably determine transcriptional start sites; direct biochemical measurements (primer extension and sequencing on primary transcripts; PolII occupancy) are necessary. EST sequence comparisons provide approximate confirmation of transcription start sites in a substantial proportion of *Chlamydomonas* transcripts (Phytozome website). In the absence of other information I provisionally accept the annotated start sites as correct. These start sites, combined with annotated splicing and proposed translational start sites, result in annotated 5′ untranslated sequences (‘5UT’).

The standard model for eukaryotic translation is the ‘scanning’ model (Kozak 1978): the 40S ribosomal subunit binds at the 5′ mRNA m7GPPP cap, then scans in the 3′ direction until the first AUG, which is the translation start codon. Location of this codon triggers joining of the 60S subunit and initiation of translation (reviewed by Hinnebusch 2011). Exceptions to this rule (skipped 5′ AUGs) may frequently be ascribed to lack of the ‘Kozak’ consensus (Kozak 1989) in inefficient initiators, which are skipped by the scanning ribosome. In some cases, a short upstream ORF (uORF) may be translated and terminated without full ribosome disengagement; provided the distance to the next AUG is not long, reinitiation can occur at a downstream AUG without rebinding to the cap. This provides the potential for regulatory mechanisms, the best-studied being yeast GCN4, where starvation effectively increases the distance the ribosome can continue scanning to reach an internal AUG (Hinnebusch 2011). Internal ribosome entry sites (IRES) are found in some viral RNAs encoding multiple polypeptides, which allow cap-independent ribosome binding and initiation; such sequences are rare or nonexistent outside of viral systems.

Transcripts in the annotated *Chlamydomonas* genome have indicated transcription start sites and 5′ untranslated sequences (‘5UT’). These annotated landmarks and the reference sequence lead to the result that ∼13,000 of the 19,526 transcript models contain one or more 5′ untranslated ATGs (Table 1). (Note: although ‘ATG’ formally refers to a DNA sequence that never sees a ribosome, since this paper is entirely based on DNA sequence I will ignore this fact in discussing ATGs ‘initiating translation.’)

**Table 1:** Annotated 5′ untranslated sequences for all annotated transcripts were extracted from the reference *Chlamydomonas* genome and analyzed for potential uORF content

	uORF Classes Present		
Number of Transcript Models	1	2	3
6438	—	—	—
769	+	—	—
821	—	+	—
6693	—	—	+
421	+	+	—
1600	+	—	+
1514	—	+	+
1270	+	+	+
Sums			
4060	+		
4026		+	
11077			+

**Figure 1:**
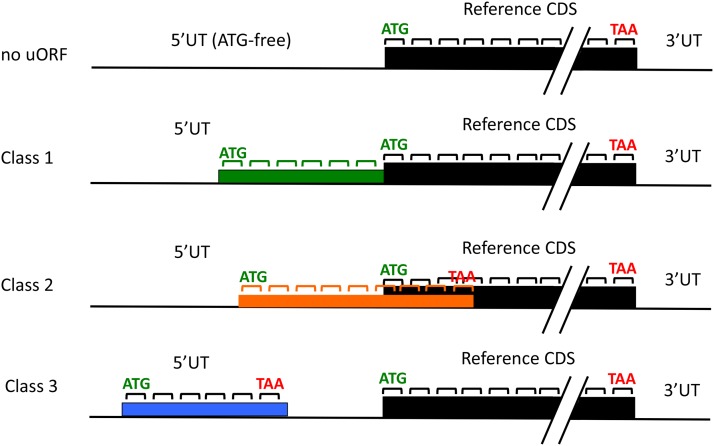
Three classes of upstream open reading frames. The 5′ untranslated sequence (5UT) can contain no ATG in any frame (top), resulting in no upstream open reading frame (‘uORF’). It can contain one or more ATGs in frame with the main coding sequence (‘Reference CDS’), without an intervening stop codon (Class 1). Class 2 is the same as Class 1 but in a different reading frame; in general the Class 2 uORF will terminate shortly after entering the main CDS out-of-frame. Class 3 initiates (in any frame) and terminates within the 5UT. Note that I consider a maximum of one Class 1 and one Class 2 uORF per transcript, although these uORFs can contain internal ATGs that could in principle initiate a ‘different’ Class 1 or Class 2 uORF. A given transcript can (and frequently does) contain multiple Class 3 uORFs, in the same or different frames. I consider all of these as separate individuals.

For schematic of classes see Figure 1. Class 1: ATG in 5UT sequence, in-frame with reference CDS, without intervening stop codon; class 2: ATG in 5UT sequence, out-of-frame with reference CDS, without intervening stop codon; class 3: ATG in 5UT sequence, with stop codon in-frame before the reference CDS. A given transcript can in principle have any number of each class. Sums: total transcripts containing at least one of the indicated class of uORFs.

These uORFs fall into three classes (Figure 1). Class 1 was in-frame with the annotated translation start site (henceforth, the ‘reference’ start), with no intervening stop codon. Thus, if translation initiated at the upstream ATG, an N-terminal extension (the uORF) would be appended to the expected reference peptide. Class 2 ATGs are out-of-frame with the reference start site, with no intervening stop codon. Initiation at class 2 ATGs thus would produce a peptide (the uORF plus frameshifted translation from the annotated CDS) lacking any protein sequence relationship to the predicted peptide product of the Phytozome transcript (the ‘reference peptide’). Class 3 ATGs initiate potential 5′ uORFs that terminate within the annotated 5UT region. A given transcript model can have examples of all three classes of uORFs (Table 1). Note that according to the default scanning ‘1st AUG’ model, class 2 and 3 uORFs should completely prevent translation of the annotated Phytozome CDS in ∼12,000 of the 19,526 transcripts, despite the fact that in many cases this CDS displays high evolutionary conservation (Merchant *et al.* 2007); the same rule would result in an obligatory N-terminal extension to numerous predicted peptides encoded by class 1 transcripts. Thus these highly abundant uORFs present a *prima facie* problem with respect to translational control and the predicted proteome.

In a broad range of organisms, ATG frequency is significantly reduced in 5′UΤ compared to CDS (Zur and Tuller 2013). In the *Chlamydomonas* annotation, the frequency of ATGs in-frame with CDS is 40% lower in 5UT than in CDS (excluding the reference initiator itself), while the frequencies of out-of-frame ATGs is 73% higher (Table 2). These departures are largely due to deviations from random expectations specifically in the CDS (where the random model is based on overall dinucleotide frequencies). Overall, though, ATG frequency in 5UT and CDS are nearly identical (0.029 *vs.* 0.028), in contrast to results in other organisms (Zur and Tuller 2013).

**Table 2:** Frequency of ATG in combined coding sequence (CDS), 5UT sequence (5UT), and expected random frequency with length-matched sequence sets with the same dinucleotide frequency as in genomic CDS or 5UT

Sequence	ATG_inframe	ATG_outframe
CDS	0.017	0.011
Rand_CDS	0.010	0.022
5UT	0.010	0.019
Rand_5UT	0.013	0.026

Frequencies are numbers detected divided by total sequence length/3. ATG_inframe: in-frame with CDS (excluding the initiator itself); ATG_outframe: out-of-frame with coding sequence. Randomized results were similar if mononucleotide frequencies were used instead of dinucleotides.

### Class 1 uORFs are longer than expected for random sequence

If uORFs have biological relevance, this could be reflected in statistical sequence differentiation from randomized controls. A ‘scrambled 5UT-ome’ was constructed by individually randomizing sequence of each 5UT sequence, thus preserving nucleotide composition but not sequence. This control set of uORFs will reflect sequence-independent consequences of the nucleotide composition and length distribution of the reference 5UTs. Because dinucleotide frequencies can vary between different genomes or different regions of the same genome, independent of single nucleotide frequencies, a second control set was constructed by determining dinucleotide frequencies in the 5UT-ome overall, and making random sequences matched for lengths and overall dinucleotide frequencies. Additional controls were derived by ‘mutagenizing’ the annotated 5UTs by randomly replacing on average one tenth, one fifth, or one half of the nucleotides in each 5UT with another nucleotide chosen based on the overall 5UT nucleotide frequency. These controls (especially the 1/10 or 1/5 mutagenesis) will lack only strongly sequence-dependent features when compared to the reference 5UTs.

**Figure 2:**
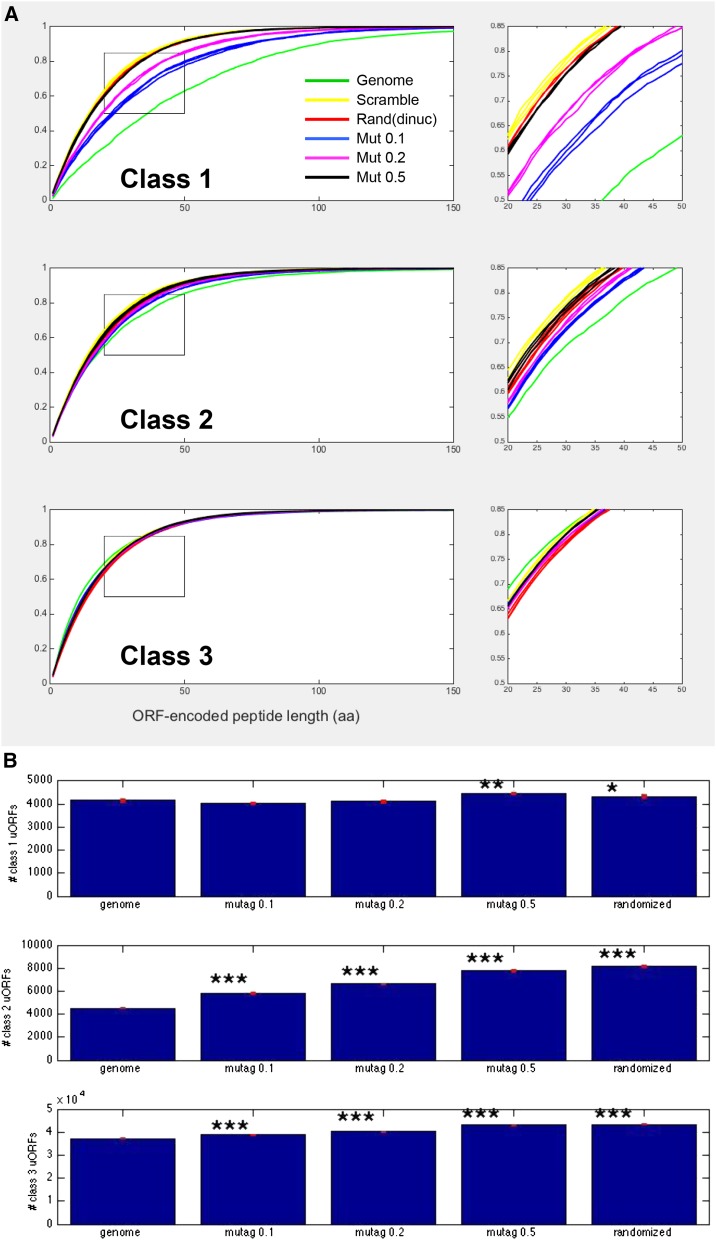
Statistics of uORF length and number in the genome, and in partially or fully randomized controls. (A) Class 1 but not Class 2 or Class 3 uORFs are longer than the random expectation. Cumulative length distribution of three classes of uORFs for the genome (green) and for randomized or mutagenized controls (three replicates of each). ‘Scramble’: each 5UT sequence was randomized (yellow); ‘Rand(dinuc)’: sequences of the same length as the real 5-UT sequences were constructed with identical dinucleotide frequencies to the overall ‘5UT-ome’ (red); Mut 0.1/0.2/0.5: the set of 5UT sequences was ‘mutagenized’ by replacing one in 10, one in five, or one in two nucleotides in each 5UT with random selections from the overall nucleotide frequency distribution of the complete collection of 5UT sequences. (Note: the randomized distribution for all classes is essentially identical to the class 3 length distribution for the actual genomic Class 3 sequences.) The indicated box in each graph is blown up at right to show high reproducibility of randomized results for the three replicates. (B) Total numbers of uORFs with and without randomization. The small red bar represents a hypothetical standard deviation based on the assumption that numbers in each category are Poisson-distributed (square root of the number observed). Stars represent *P*-values for a *t*-test comparing each randomization to the genome, using these standard deviations: * *P* < 0.05; ** *P* < 0.01; *** *P* < 0.001). Randomizing by scrambling (shown) or by dinucleotide frequencies gave very similar results.

Results of these comparisons with respect to number and length of uORFs were very clear, and strikingly different for the three classes. For classes 2 and 3, uORF lengths were essentially identical for the reference and the random or mutated controls (Figure 2A). In contrast, class 1 uORFs from the genome were significantly longer than in any of the controls. Mutation of one in ten nucleotides was almost as effective at eliminating sequence dependence as complete randomization, suggesting a high degree of sequence dependence in the real sequence. The numbers of class 1 and class 3 uORFs were approximately similar in reference and controls (Figure 2B); however, class 2 uORFs doubled in abundance in the randomized control compared to the reference. Note that the random expectation is that class 2 should be twice as abundant as class 1, since there are two ‘wrong’ frames and one ‘right’ frame.

The effectiveness of ‘mutagenesis’, even at a 10% substitution rate, at reducing lengths of class 1 uORFs was primarily due to gain of internal termination codons; this mechanism preferentially removed longer class 1 uORFs from the compilation (by conversion to class 3). Class 1 uORFs were also lost or shortened due to loss of the initiator ATG. New class 1 uORFs appeared upon mutagenesis, but these were generally shorter, accounting for decrease in length with no change in overall number.

These results suggest sequence-dependent constraints that (1) preserve class 1 uORFs at significantly longer than expected by chance and (2) suppress the numbers of class 2 uORFs to about half the level expected by chance. Class 3 uORF numbers and lengths are strikingly well predicted by nothing more than 5UT nucleotide composition (mono- or dinucleotide-based) and length distribution, and thus exhibit no sequence dependence detectable by this bulk approach. The close statistical correspondence of the genomic class 3 uORFs and those constructed from randomized sequence suggests that most class 3 uORFs are effectively neutral sequence [though statistically significant increases in class 3 uORF numbers in randomized controls (Figure 2B) suggest purifying selection over at least a subset]. The suppression of class 2 uORF number relative to random controls suggests that class 2 uORFs, in contrast, are subject to significant purifying selection. This could be understood based on the scanning model for translation initiation since initiation from a class 2 AUG would block even post-termination reinitiation at the reference AUG. This is because scanning is probably generally (though perhaps not exclusively) unidirectional (Hinnebusch 2011), and class 2 termination occurs 3′ to the reference initiation site (Figure 1). Thus class 2 AUGs could be particularly damaging to expression of the main CDS.

### A Chlamydomonas Kozak consensus

These observations raise issues with respect to the standard translation model. Since the reference initiation ATG generally starts translation of a long peptide, which is frequently conserved across species, it is highly unlikely that Class 2 and Class 3 ATGs are exclusive sites of initiation. This suggests a high level of selectivity, since nearly two thirds of transcripts are assigned class 2 and/or class 3 uORFs—so if the reference ATG is in fact the one used *in vivo*, multiple 5′ ATGs must fail either initiation or ribosome disengagement in a majority of transcripts—thus, the scanning model would be the exception rather than the rule.

There are two obvious escapes from this problem. The simplest is if the annotated transcription start site is misplaced at a position 5′ of the real start. For a substantial subset of Phytozome gene models, there is evidence from EST sequence that the transcript does indeed cover some or all of the proposed 5UT, so this is unlikely to be the entire explanation.

**Figure 3:**
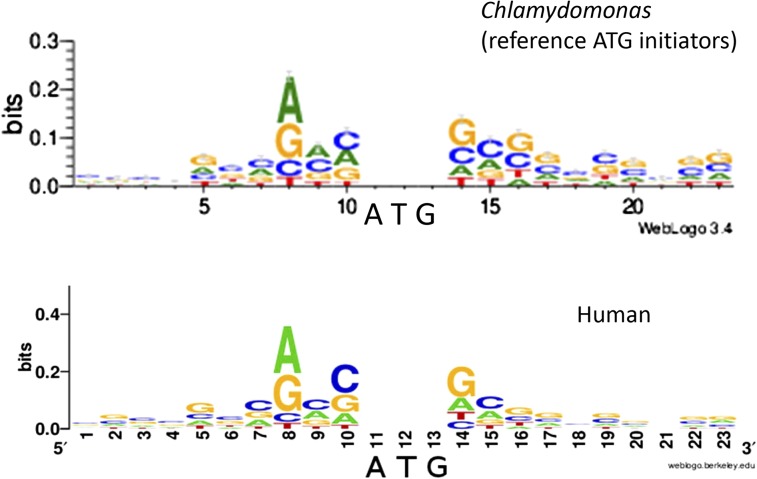
Kozak-like consensus sequence around *Chlamydomonas* reference initiator ATGs. All 19,228 sequences were fed to the online WebLogo tool (http://weblogo.threeplusone.com) (Crooks *et al.*, 2004). A comparable plot for human mRNAs was downloaded from Wikipedia (https://en.wikipedia.org/wiki/ File:Human_Kozak_context._Version_2).

Another escape would be strong sequence constraints suppressing initiation at uORFs. This is a known mechanism in other eukaryotes, where absence of a ‘Kozak’ consensus sequence can allow skipping of a 5′ AUG (Kozak 1989; Hinnebusch 2011). Using the Weblogo calculation (Crooks *et al.* 2004) with the complete set of *Chlamydomonas* reference initiator ATGs yielded a consensus with striking similarity to the Kozak consensus found for human mRNAs (Figure 3).

**Figure 4:**
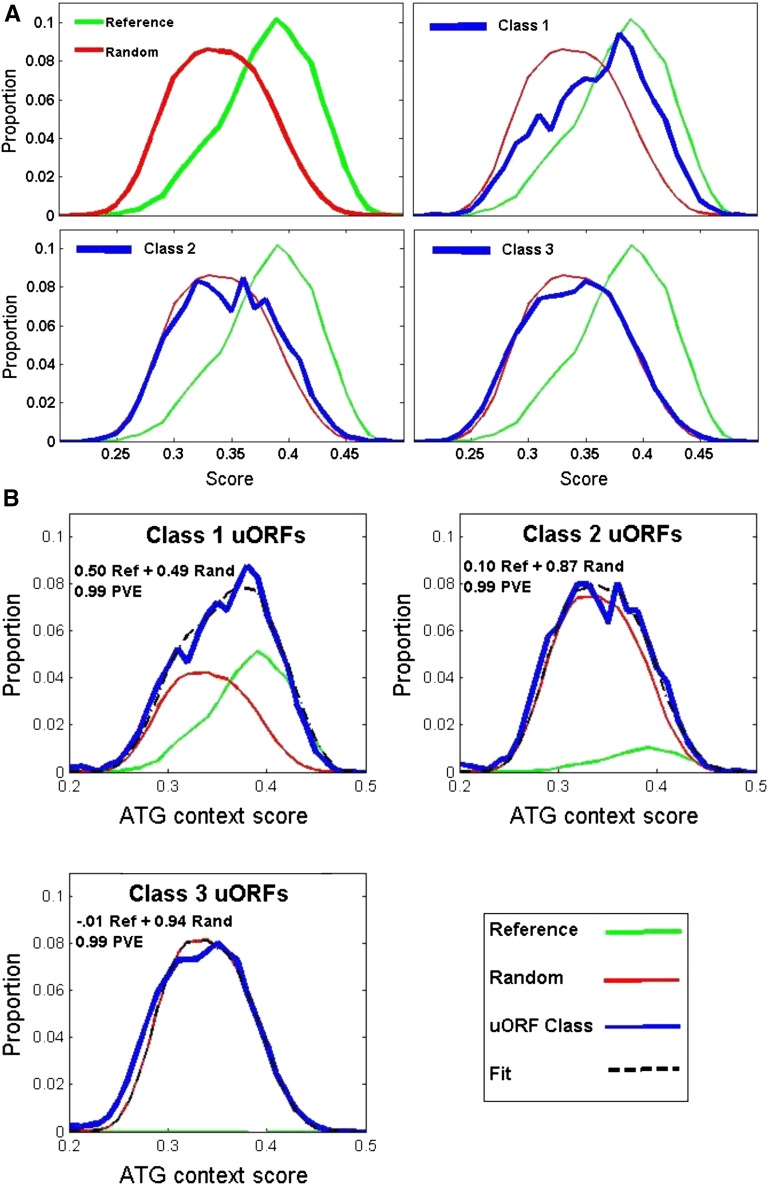
Class 1 uORFs, but not Class 2 or Class 3 uORFs, show significant agreement to the Kozak consensus. (A) Reference, random, and uORF agreement with the consensus. A ‘Kozak score’ was defined as the sum of bits corresponding to the observed nucleotides at each position surrounding the ATG (Figure 3). Top left: comparison of scores of reference initiator ATGs (green) to scores of 100,000 random sequences composed with the nucleotide frequency of 5UT (red). Remaining panels: uORFs (blue) compared to reference and random distributions. (B) Projection of uORFs onto space spanned by reference initiators and random sequence. Define Matrix A = [distribution of reference;distribution of random]; vector C = distribution of uORF; then [c1 c2] = (A^T^A)^-1^ A^T^C gives the least-squares best-fit solution for c1 × reference distribution + c2 * random distribution ≈ C (Strang 2009). The best fit is the dotted black line; weights and proportion of variance explained (PVE) are indicated.

This consensus allowed construction of an ATG context ‘Kozak’ score based on agreement with the consensus and the information content of the position. The distribution of Kozak scores for reference ATGs to scores for 100,000 random sequences composed with nucleotide composition of the overall 5UT-ome of *Chlamydomonas*. There was a clear separation between unimodal score peaks (Figure 4A). The result was the same with randomization by mono- or dinucleotide frequencies.

The distribution of scores for reference initiators was also separated from randomized sequence at the nucleotide distribution of CDS, and from the distribution of scores for internal ATGs (3′ to the reference). Interestingly, the ATG immediately 3′ to the reference ATG (but not further-internal ATGs) had a distribution of scores slightly but significantly lower than the random expectation, possibly suggesting the importance of discriminating the first from the second potential initiator (figures generated by supplemental MATLAB code).

### Distribution of Kozak consensus scores suggests that some Class 1 uORFs may be translated

Class 2 and Class 3 ATGs had a distribution of scores nearly identical to the random sequence control (Figure 4A). Thus, if the Kozak consensus enhances initiation efficiency, many class 2 and 3 ATGs may be skipped in favor of the downstream reference ATGs.

Class 1 ATGs had a very different distribution of Kozak consensus scores, which resembled a bimodal mixture of score distributions similar to the randomized control, and similar to the reference ATGs. A simple linear algebra calculation (projection of the data onto a 2-dimensional space spanned by the randomized control and the reference ATG distributions) yielded the optimal mixture: a 51:49 combination of these distributions yielded a very good fit to the class 3 distribution (99% of variance explained by this linear fit) (Figure 4B).

This observation suggests the hypothesis that class 1 uORFs are heterogeneous. Half of them might be inefficiently translated, thus resembling the Class 2 and Class 3 uORFs. Half, on the other hand, might be translated either as alternative or as the exclusive *in vivo* initiations. Such initiation would result in a peptide with an N-terminal uORF fused to the reference peptide.

### Test of translation-dependent evolutionary selection on class 1 uORFs

If class 1 uORFs are translated and the resulting N-terminal extensions are under evolutionary constraint, then the N-terminal sequence could extend the alignment of the predicted peptide, when compared to other organisms. *Volvox* is a multicellular species with a recent common ancestor with *Chlamydomonas* (Herron *et al.* 2009). Many *Chlamydomonas* peptides have highly similar orthologs in *Volvox* (Prochnik *et al.* 2010). However, neutral nucleotide sequence divergence between *Volvox* and *Chlamydomonas* is >50%, based on substitution rates at neutral positions in highly conserved proteins (unambiguously alignable without gaps: actin, tubulin, CDKB). Thus sequence not under selection for its protein coding potential should rapidly lose any recognizable BLASTP (protein) similarity, due to divergence and especially to fragmentation from gain of termination codons and loss of potential initiator ATGs. (Figure 2A showed that a 50% divergence rate was equivalent to full randomization for completely eliminating enhanced lengths of class 1 uORFs.) In contrast, if a sequence is translated and the peptide product under selection, then BLASTP similarity will be retained.

**Figure 5:**
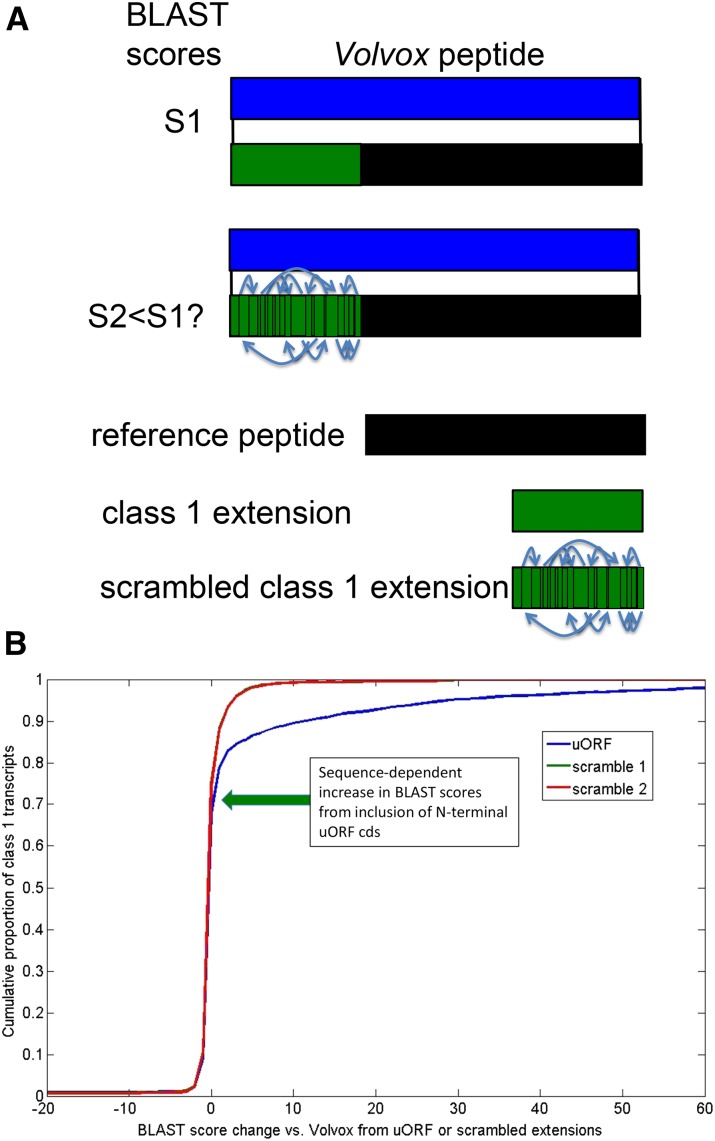
Many class 1 uORFs encode evolutionarily conserved N-terminal extensions. (A) Schematic of the test. BLASTP alignments to the *Volvox* proteome were carried out for each class 1 transcript using four different versions: the reference transcript; the reference N-terminally extended by the class 1 uORF; and the reference transcript N-terminally extended by scrambled versions of the class 1 uORF peptide. Sequence-dependent BLASTP score improvement (S1 > S2, S2 ≅ S3) was taken to argue for evolutionary conservation of class 1 CDS. (B) The differences between maximal BLASTP scores of the reference peptide and the N-terminally extended versions (class 1 uORF peptide, or the scrambled uORF peptide) for all class 1 transcripts are plotted as cumulative distributions. The divergence of the uORF peptide from the scrambled versions at a score of >4 and about 70%–75% of transcripts (divergence point marked with green arrow) indicates sequence-dependent score increase (that is, score increase specific to the uORF and not to scrambled versions) in 25%–30% of class 1 transcripts.

BLASTP scores of the *Volvox* proteome were determined against *Chlamydomonas* reference peptides, reference peptides with class 1 uORF N-terminal extensions, and controls of reference peptides with class 1 uORF extensions that were scrambled at the level of predicted peptide (two scrambling replicates). This scrambled control takes into account possible BLASTP score improvement due to simple-sequence features (*e.g.*, poly-Pro aligning similarly with and without scrambling with Pro-rich N-termini in subject proteins). The test statistic was the maximum BLASTP score due to the uORF extension compared to scrambled controls. (This score is in units of bits, which are logarithmic; thus the arithmetical difference in scores is an appropriate indicator of differential effect of the uORF). In many cases the real uORFs, but not the scrambled controls, increased the score by up to many hundreds of bits. Quantitative comparison of cumulative results suggests that at least 25–30% of class 1 uORFs are under selection for translated sequence content (Figure 5). This is likely a lower bound for the proportion of class 1 uORFs that are translated *in vivo*: first, because many proteins lack BLASTP-detectable similarity at their N-termini; second, because the comparable stretch in the *Volvox* annotation might have also been incorrectly assigned to an untranslated uORF.

**Figure 6:**
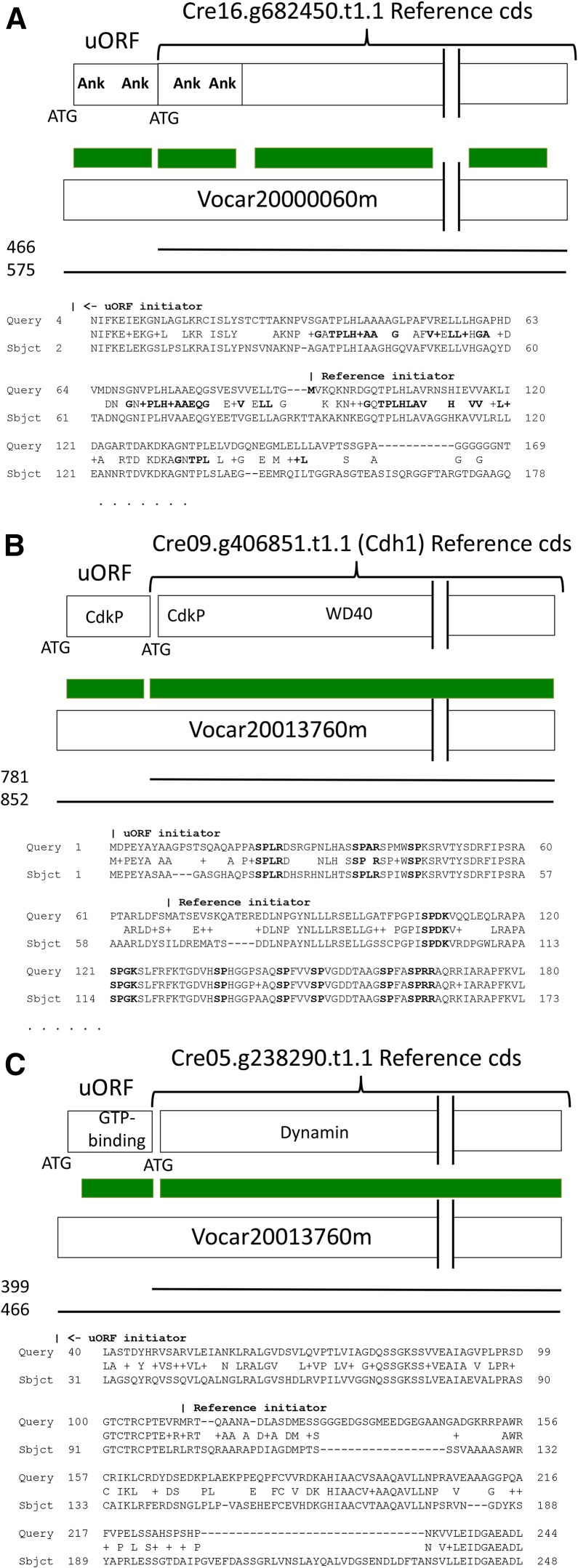
Examples of BLASTP score improvement by class 1 uORF N-terminal extensions. The N-terminal sequence of the best *Volvox* BLASTP hit to the reference CDS is shown, starting from the indicated ‘Reference initiator,’ along with the extended alignment from the class 1 uORF starting at the ‘uORF initiator.’ Regions of alignment are sketched in green and BLASTP scores indicated at left. (A) Ankyrin repeat-containing protein. Two ankyrin repeats (bold in alignment below) are found in the N-terminal extension, and two more in the reference CDS. (B) Cdh1. Cdh1 is known to be regulated by cyclin-dependent-kinase phosphorylation (Zachariae *et al.*, 1998) (minimal consensus S/T-P; extended consensus S/T-P-x-R/K). Three out of seven such sites are in the class 1 uORF N-terminal extension. (C) Dynamin-homologous protein, with characteristic GTP-binding domain of dynamins encoded in the class 1 uORF extension.

A few examples of the aligned sequences resulting from these BLASTP comparisons are presented in Figure 6. It is clear in these cases that the uORF encodes evolutionarily conserved sequence, relevant to the function of the peptide. The data comprise a continuous series from such obvious cases to addition of only a few amino acids, with marginal or no effect on BLASTP scores.

A complete tabulation of the results of the BLASTP analysis is provided in Table S1.

### Correlation of high Kozak consensus score and probable in vivo translation for Class 1 uORFs

**Figure 7:**
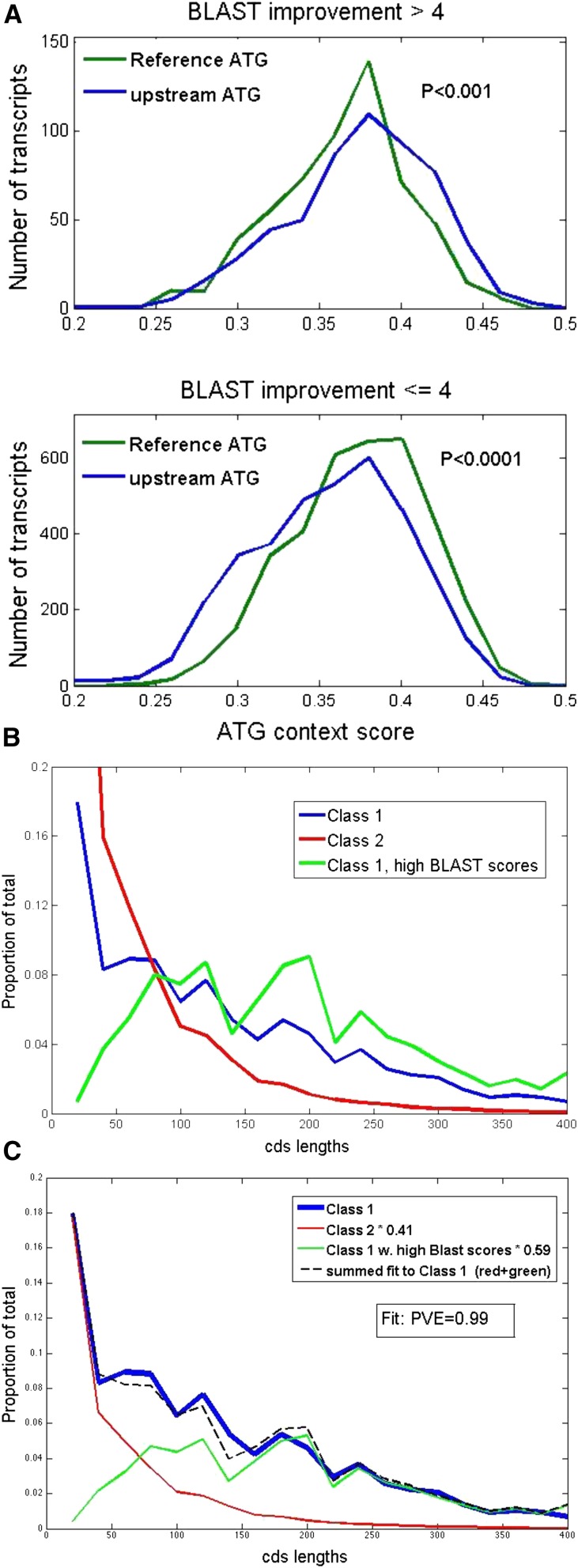
Class 1 uORFs with high BLASTP scores have high ‘Kozak’ scores and long CDSs. (A) Above: Kozak scores for the class 1 uORF ATG and the reference ATG, for all transcripts in the high BLASTP-improvement score class (see Figure 5). Below: Kozak scores for the same ATGs in the low BLASTP improvement score class. Insets: probability by *t*-test in the high BLASTP class that reference ATGs have average scores greater than or equal to uORF ATGs (above), probability in the low BLASTP class that reference ATGs have average scores less than or equal to uORF ATGs (below). (B) High BLASTP-score class 1 uORFs are longer than average class 1 uORFs. (C) The length distribution of the total pool of class 1 uORFs can be accounted for as a 0.59:0.41 sum of the high BLASTP-score subset distribution and the Class 2 uORF distribution, by least-squares fitting as in Figure 4B.

An apparent bimodal distribution of Kozak consensus scores among class 1 uORFs led to the suggestion above that the higher-scoring class 1 uORFs might be preferentially translated. Figure 7A shows Kozak score distribution of the class 1 ATG and the reference ATG from the upper ∼25% of BLASTP improvement, compared to the remainder. The high-scoring cases had Kozak scores indistinguishable from overall reference ATG initiators, and interestingly had significantly better scores than the reference initiators from the same set of transcripts. The lower 75% were essentially indistinguishable from the bulk (compare Figure 7A to Figure 4A, top right).

This finding supports the ideas that the Kozak consensus is relevant to translation efficiency, and that many class 1 uORFs are translated *in vivo*, either as alternative or as the sole initiation codons.

Class 1 uORFs are significantly longer on average than either class 2 uORFs, or class 1 uORFs from ‘mutagenized’ or randomized sequence (see above). This length effect was markedly enhanced for the class 1 uORFs in the upper 25% of BLASTP improvement (Figure 7B). If class 1 uORFs are not translated, there is no obvious reason for any length difference compared to class 2 uORFs, since they differ only in translational frame relative to the reference (Figure 1). The total class 1 CDS length distribution could be closely approximated as a linear combination of the class 2 distribution and the high BLASTP-scoring class 1 distribution with ∼40:60 mixture (Figure 7C). This split is similar to the 49:51 split in the fit to Kozak consensus scores; both results suggest that around half of class 1 uORFs are translated efficiently. (Note that if the class 1 uORFs are a heterogeneous 50:50 mixture of neutral and functional sequences, then considering only the neutral class, the lengths and lack of sensitivity to mutagenesis and randomization becomes very similar to class 2; their numbers become just about half that of the class 2 uORFs, as expected given three reading frames.)

### Weighted predictions for translation of class 1 uORFs

The results above suggest that approximately 50% of class 1 uORFs are alternative or exclusive sites of *in vivo* initiation. Where a strong improvement in BLASTP score could be detected by including the uORF N-terminal extension, these transcripts can be identified directly (Figure 5 and Figure 6). Such cases are a minority, though, and it would be desirable to have a quantitative estimate of the probability of translation initiation for all ∼4000 class 1 uORFs.

**Figure 8:**
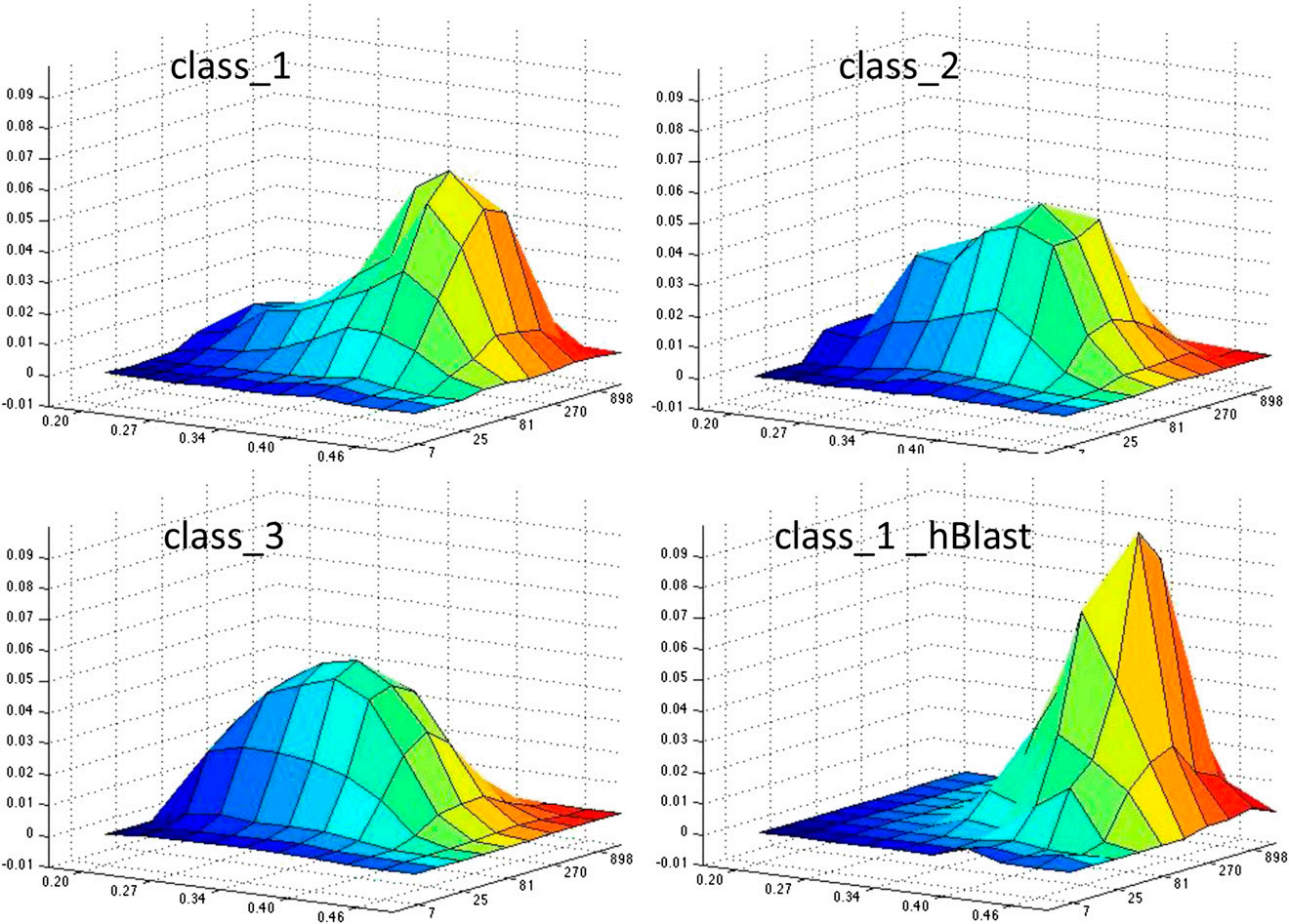

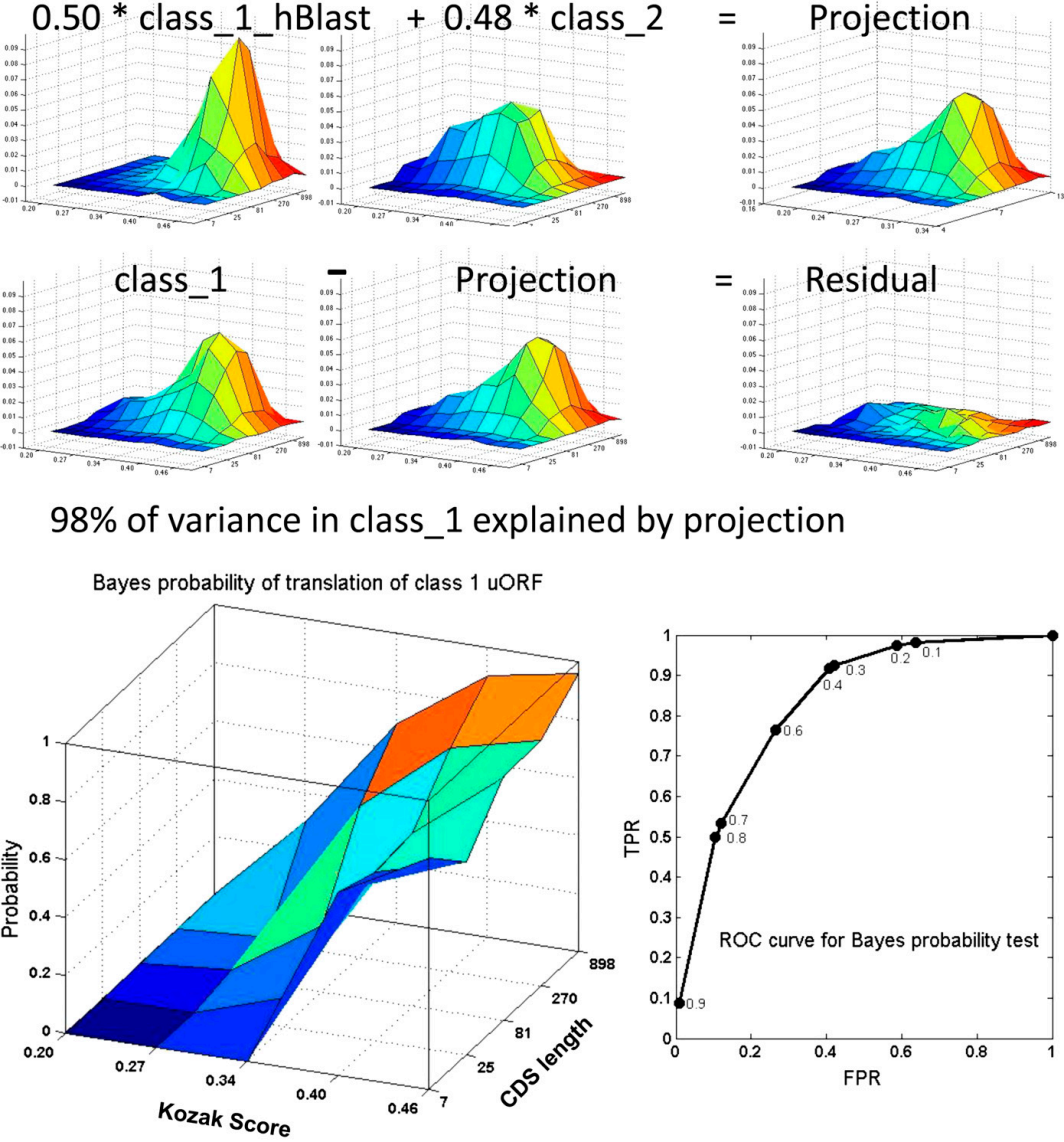
Two-dimensional accounting for uORF variability. (A) Surface of proportion of uORFs over a two-dimensional grid of Kozak score (exponentiated) and log CDS length. These transformations were chosen because the Kozak score is based on an essentially logarithmic information scale (Figure 3), whereas CDS lengths can be conceptualized as due to exponential decay from finite probability of hitting a stop codon (class 3) or the beginning of the CDS (classes 1 and 2). Classes 2 and 3 have broad peaks with low Kozak scores (left axis) and shorter CDSs (right axis). The high BLASTP-score subclass of class 1 uORFs (lower right) has a sharp peak at high Kozak score and longer CDSs. The complete pool of class 1 uORFs appears heterogeneous, with a peak similar to the high BLASTP subset and a shoulder similar to the distributions of classes 2 and 3. (B) Least-squares accounting for the two-dimensional class 1 uORF distribution as a sum of 0.50 high BLASTP class 1 uORF subset and 0.48 class 2 uORF (matrix calculation as in Figure 4B); 99% of variation is explained. (C) A Bayesian test for translation of class 1 uORFs. Call T the event of translation; K a given Kozak score; L a given length. We want to know P(T | K&L). Bayes theorem says this is equal to: P(K&L | T) × P(T) / [P(K&L | T) × P(T) + P(K&L | ∼T) × P(∼T)]. Assuming ∼50:50 split of translated and untranslated class 1 uORFs (Figure 4C, Figure 7C, and Figure 8B) [*i.e.*, P(T) = P(∼T)], this simplifies to P(T | K&L) = P(K&L | T) / [P(K&L | T) + P(K&L | ∼T)]. K&L specifies a point on the grid; P(K&L | T) is proportional to the height of the high-BLASTP surface over this point, while P(K&L | ∼T) is proportional to the height of the class 2 surface over this point. So this ratio calculated over the whole grid provides a probability estimate. This surface is graphed on the left. On the right is the ROC curve given this surface, for varying cutoffs of nominal Bayesian probability, with the assumption that all translated class 1 uORFs will have sequence properties similar to the high BLASTP score positive training set, while all untranslated class 1 uORFs will be similar to (the presumably untranslated) class 2 uORFs negative training set.

Class 1 uORFs that are more likely to be translated have two sequence features (independent of *Volvox* alignments): the uORF CDS is longer, and the Kozak consensus is stronger. Neither feature is quantitatively strong enough to form a digital classifier. The two-dimensional differentiation between uORF classes 1, 2, and 3, and the ‘high BLASTP’ subclass of uORF class 1 was a stronger separator (Figure 8A). The surface for class 1 could be almost exactly modeled as a 50:50 split between the class 1-high BLASTP subclass (representing efficiently translated uORFs) and class 2 (representing presumably poorly translated uORFs) (Figure 8B). This allowed construction of a simple Bayesian test for the likelihood that a class 1 uORF is translated based on its CDS length and its Kozak score, using as training sets the high BLASTP class 1 subset (positive examples) and class 2 uORFs (negative examples). The strength of discrimination is illustrated in Figure 8C; the receiver–operator characteristic (ROC) curve shows the false positive/true positive relationship for various probability cutoffs. While this test is still certainly not definitive in any individual case, it does provide an empirically based estimate of the probability of translation in the complete set of class 1 uORFs (Table S1). In advance of more definitive empirical evidence, these probabilistic classifications may be helpful as an adjunct to the (necessarily) digital summary in Phytozome that some sequence ‘is’ or ‘is not’ part of the CDS.

The conclusion that a substantial proportion of class 1 uORFs is translated is based on multiple comparisons and tests that are entirely independent: length distributions of class 1 CDS compared to classes 2 and 3, and to randomized controls; Kozak score distributions of the same set, also compared to reference ATGs; BLASTP improvement in comparison to the *Volvox* proteome. The estimated 50:50 split is also based on projection of class 1 uORF data onto multiple distinct subspaces: spanned by Kozak scores of reference ATGs and random sequence; and spanned by CDS length of the high BLASTP class 1 subset and class 2 uORFs, alone or in two-dimensional combination with Kozak scores.

### A large majority of reference ATGs are likely contained in CDS

The analysis so far was focused on the question of whether the reference ATG or a more 5′ ATG was a more likely site of translation initiation. A converse question can be asked: could some reference ATGs in fact themselves begin class 1 uORFs, with the authentic *in vivo* start codon being one annotated as internal?

**Figure 9:**
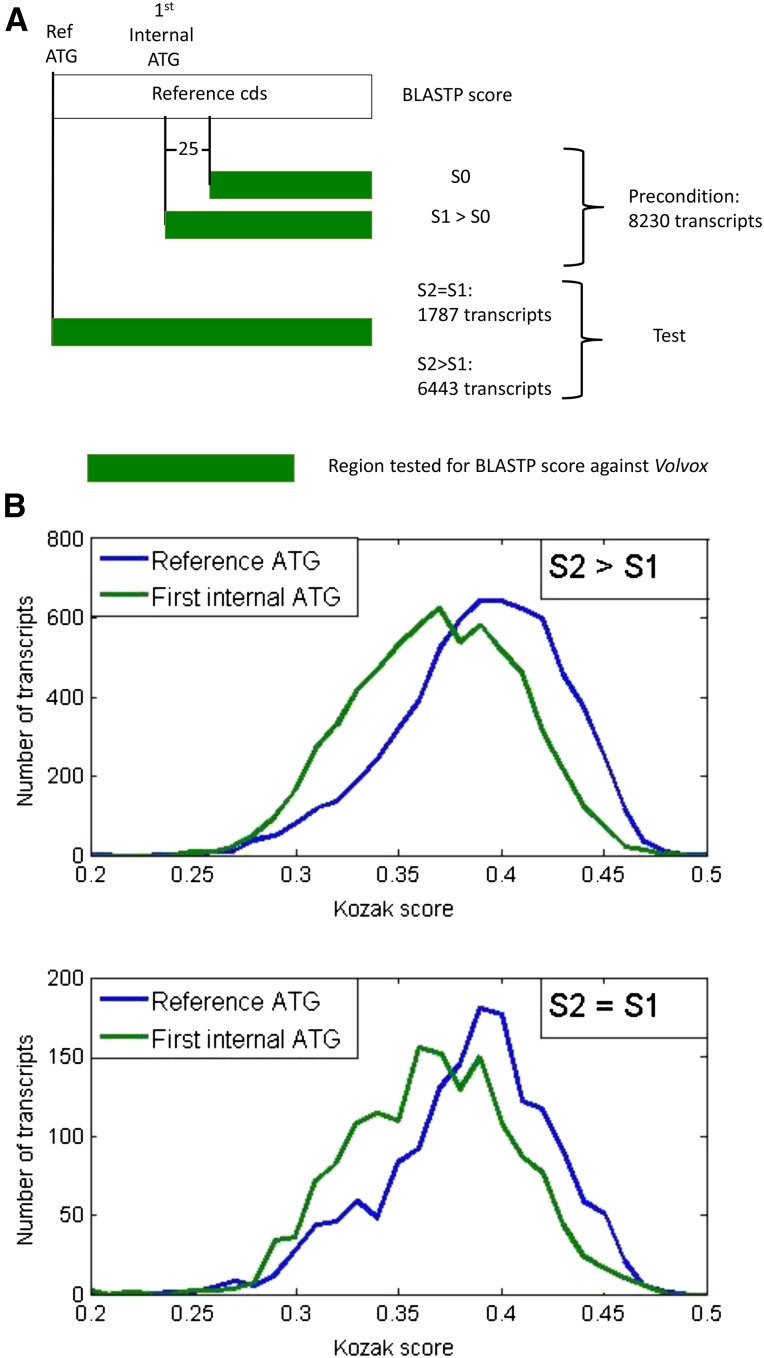
Test for reference ATG being in CDS. (A) Call the reference ATG the ‘first’ ATG, and the succeeding in-frame CDS-internal ATG the ‘second’ ATG. In many coding sequences there will be BLASTP similarity to a *Volvox* peptide, aligning from 25 amino acids C-terminal to the second ATG to the end of the CDS, with score S0 (green bar indicates region of alignment). In a subset of these cases this alignment and BLASTP score will increase if the query for alignment begins exactly at the second ATG (rather than 25 amino acids to the C-terminus). This precondition (score S1 > S0) for the test is met in 8230 transcripts. In such cases, if the first ATG is the genuine initiator, it is quite likely that the score will increase to S2 > S1 if the BLASTP query additionally includes the segment encoded between the first and second ATG. In contrast, if the first ATG is not translated, then the score will almost surely not increase (S2 = S1), since conservation to *Volvox* should require selection based on evolutionarily conserved translation to peptide. In 78% of transcripts fitting the precondition S1 > S0, we observed S2 > S1, setting an upper bound of 22% for the frequency of reference initiators not actually translated. (B) Comparison of Kozak scores for the first (reference) ATG to those for the second ATG, in the two cases indicated in part A. In both cases the reference ATG population had significantly higher Kozak scores than second ATG, suggesting that the reference initiator is a more probable *in vivo* site of translation initiation even in the cases below failing the BLASTP score test.

Another BLASTP comparison to *Volvox* provides a test. (In the following, for simplicity, I will call the reference ATG the ‘first’, and the succeeding ATG in the 3′ direction the ‘second’). In this comparison, I first determined the subset of transcripts for which there was a detectable *Volvox* BLASTP hit for which the maximum score was dependent on sequences immediately 3′ to the second ATG. There were 8320 such transcripts. In 78% of this set of transcripts, the segment between the first and the second ATG contributed further to the BLASTP score (Figure 9A), which would be unexpected if the first ATG was not in the translated product. This could be taken to imply that as many as 22% of ‘reference’ ATGs are not, in reality, part of CDS. Arguing against this idea, though, Kozak scores were on average higher for the first than for the second ATG (*P* < <<0.001 by *t*-test for both comparisons) independent of BLASTP results (Figure 9B). Therefore, at least 78%, and likely a higher proportion, of the reference ATGs are actually within CDS.

### Downstream open reading frames

**Figure 10:**
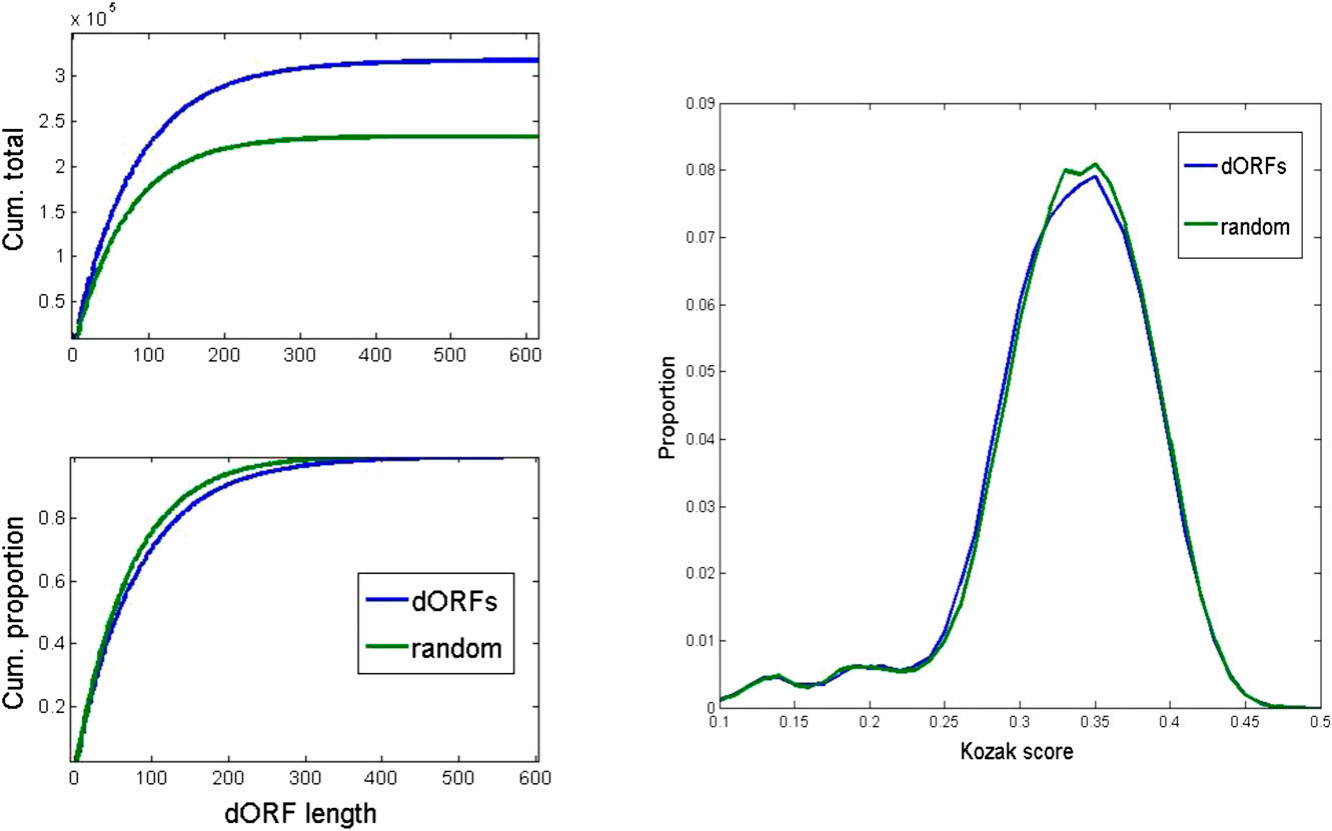
Downstream open reading frames. Open reading frames within annotated 3′ UTR sequences (downstream open reading frames or dORFs) were compared to dORFs from a randomized control with the same overall nucleotide composition and same length distribution. Top left: cumulative numbers; bottom left: cumulative proportions. Right: distribution of Kozak scores for real and random dORF ATGs.

The high prevalence of class 3 uORFs in the annotation suggests the possibility of translation reinitiation, so that if a class 3 uORF is translated this does not block translation of the main reference CDS. If such reinitiation is common, it raises the possibility that open reading frames in the 3′ untranslated region (downstream ORFs or ‘dORFs’) might similarly be translated after termination of the reference CDS. There are massively abundant open reading frames in the annotated 3′ untranslated regions – ∼300,000 in all (Figure 10, top left), mostly less than 100 nt in length. A randomized control has about half as many dORFs. The distributions of dORF lengths and Kozak scores are identical between the real and randomized data. These observations do not strongly suggest that dORFs are translated in great abundance, although the increased number compared to the randomized control suggests some structure to this population.

## Discussion

### The translational fates of abundant uORFs in the annotated Chlamydomonas genome

The annotated *Chlamydomonas* genome contains a very high number of uORFs in annotated 5UT. Since a large majority of these uORFs are upstream of a reference CDS that is evolutionarily conserved, it is very likely that the uORFs do not fully block translation of this reference.

The following possibilities may explain the uORFs. (1) The transcription start site is incorrectly annotated in a majority of *Chlamydomonas* transcripts, and transcripts in fact generally initiate hundreds of nucleotides 3′ of the annotated position. This is logically possible; however, it is contradicted by EST evidence in the case of a substantial number of transcripts (Phytozome website). (2) The uORFs are not sites of translation initiation, due to sequence constraints (such as the Kozak consensus) resulting in their inefficient use. Our data support this possibility for class 2 and class 3 uORFs, and for about half of the class 1 uORFs. (3) The uORFs are sites of translation initiation, but do not interfere with translation of the reference CDS. This is our interpretation for about half of the class 1 uORFs, which likely encode translated N-terminal extensions to the reference CDS. It is an interesting possibility that class 3 uORFs are translated. Such translation can be compatible with translation of downstream AUGs (as in GCN4; Hinnebusch 2011); the GCN4 case also shows clearly that this situation can allow for regulated translation initiation. (Class 2 uORFs are likely not suitable for this mechanism, assuming that scanning is largely 3′-unidirectional, as by the time of translation termination the ribosome would be 3′ of the reference initiator.) Such mechanisms, if they exist, would likely not impose significant length or sequence constraints on class 3 uORFs, since the relevant feature might simply be having some number or density of ‘decoy’ uORFs—the sequence content might be irrelevant.

### Functional consequences of N-terminal extensions

The class 1 uORFs vary tremendously in length, and it is likely that cases where only a few residues are missed at the N-terminus in the reference annotation will frequently have rather minor functional consequences. Also, protein N- and C-termini are probably more likely to be poorly folded than other regions (perhaps explaining why terminal epitope tagging so frequently is permissive for protein function). There are many cases, though, where functional consequences from exclusion of the class 1 uORF N-terminal extension are likely quite substantial (a few clear examples, selected from many, are given in Figure 6).

Protein N-termini can contain relatively unstructured ‘addressing’ sequences for post-translational modification and/or subcellular localization, such as signal sequences for secretion or organellar transport; these would likely be undetected by BLASTP analysis. A different kind of consequence comes in searching for causative mutations following random mutagenesis: even a small, potentially unstructured N-terminal extension due to a uORF can be the site of a chain-terminating null mutation, which can go completely unrecognized if the uORF is not annotated as a possible contributor to CDS. In fact, we found just such a case in a screen for latrunculin B-sensitive mutants: a one-nucleotide deletion in an annotated 5UT region resulted in a strong mutation in a specific molecularly identified complementation group (M. Onishi *et al*. 2015). This finding was a major motivation for carrying out the present study; the deletion is, in fact, in a class 1 uORF. This ORF has a calculated probability of being translated of 0.90 by the Bayesian test described above (Figure 8C). Thus, this mutation is very likely an early chain terminator, consistent with other genetic results indicating that it produces a null allele.

### Genome annotation is probabilistic

It is a subtle problem that a sophisticated data presentation such as the *Chlamydomonas* annotated genome (Blaby *et al.* 2014) necessarily requires selection of a single one of a large number of alternatives; the most probable on some accounting is presumably selected, and serves as the sole presented version. However, the reasoning behind producing the object will generally be probabilistic and based substantially on statistical evidence. This was indeed the case for assignment of reference initiator ATGs in the *Chlamydomonas* annotation (M. Stanke, personal communication). A more accurate (though pedantic) description than ‘finding needles in a haystack’ (Blaby *et al.* 2014) might be ‘finding the most probable needles in a haystack that surely contains at least some.’ Unfortunately, gene models are amenable to neither the calculation nor graphical presentation of error bars, and certainly, most end users desire to work with ‘the answer,’ not with some probabilistically graded series of alternatives.

While this detailed examination of uORFs did uncover some issues and problems, the results suggest that for the most part, the reference initiating ATGs are the authentic *in vivo* initiators. It is encouraging that a largely computational assignment of the complex biochemical events of mRNA production and translation can be correct in a large majority of cases. The explicitly probabilistic evaluation of likely sites of *in vivo* translational initiation presented here may be useful in consideration of the *Chlamydomonas* proteome, at least until there are sufficient data from proteomics and other approaches to settle the matter definitively.

## Supplementary Material

Supporting Information
