# Supplementary material for: Tying Down Loose Ends in the Chlamydomonas Genome: Functional Significance of Abundant Upstream Open Reading Frames
Source: G3 (Bethesda). 2015 Dec 23;6(2):435–46. doi: 10.1534/g3.115.023119 (PMC4751561; doi:10.1534/g3.115.023119)
Supplement: Supporting Information [file supp_6_2_435__index.html]

Tying Down Loose Ends in the Chlamydomonas Genome: Functional Significance of Abundant Upstream Open Reading Frames — Supporting Information 

# Tying Down Loose Ends in the *Chlamydomonas* Genome: Functional Significance of Abundant Upstream Open Reading Frames

## Supporting Information for Cross, 2016

**Files in this Data Supplement:**

- File S1 - MATLAB code. (.zip, 36 KB)
- Table S1 - Results of the BLASTP analysis (.xls, 7382 KB)
